# Unexpected Heterogeneity of Newly Diagnosed Multiple Myeloma Patients with Plasmacytomas

**DOI:** 10.3390/biomedicines10102535

**Published:** 2022-10-11

**Authors:** Martin Stork, Sabina Sevcikova, Tomas Jelinek, Jiri Minarik, Jakub Radocha, Tomas Pika, Lenka Pospisilova, Ivan Spicka, Jan Straub, Petr Pavlicek, Alexandra Jungova, Zdenka Knechtova, Viera Sandecka, Vladimir Maisnar, Roman Hajek, Ludek Pour

**Affiliations:** 1 Department of Internal Medicine, Hematology and Oncology, University Hospital Brno and Faculty of Medicine, Masaryk University, 625 00 Brno, Czech Republic; 2 Babak Myeloma Group, Department of Pathophysiology, Faculty of Medicine, Masaryk University, 625 00 Brno, Czech Republic; 3 Department of Hematooncology, University Hospital Ostrava and Faculty of Medicine, University of Ostrava, 708 00 Ostrava, Czech Republic; 4 Department of Hemato-Oncology, University Hospital Olomouc and Faculty of Medicine and Dentistry, Palacky University Olomouc, 779 00 Olomouc, Czech Republic; 5 4th Department of Medicine—Hematology, Charles University Hospital and Faculty of Medicine, 500 05 Hradec Kralove, Czech Republic; 6 Institute of Biostatistics and Analyses, Ltd., 625 00 Brno, Czech Republic; 7 1st Medical Department—Clinical Department of Hematology of the First Faculty of Medicine and General Teaching Hospital, Charles University, 128 08 Prague, Czech Republic; 8 Department of Internal Medicine and Hematology, University Hospital Kralovske Vinohrady, 100 34 Prague, Czech Republic; 9 Hematology and Oncology Department, Charles University Hospital, 323 00 Pilsen, Czech Republic

**Keywords:** multiple myeloma, risk factors, survival, plasmacytomas

## Abstract

In multiple myeloma (MM), malignant plasma cells infiltrate the bone marrow. In some cases, plasma cells migrate out of the bone marrow creating either para-skeletal plasmacytomas (PS) or infiltrating soft tissues as extramedullary plasmacytomas (EMD). The aim of this study was to define risk groups in newly diagnosed MM (NDMM) patients with PS and EMD plasmacytomas. In total, 523 NDMM patients with PS plasmacytomas and 196 NDMM patients with EMD plasmacytomas were diagnosed in the Czech Republic between 2004 and 2021 using modern imaging methods. Patients’ data were analyzed from the Registry of Monoclonal Gammopathies of the Czech Myeloma Group. In NDMM patients with PS plasmacytomas, we found a subgroup with <5% of bone-marrow plasma cells to have the best prognosis (mPFS: 58.3 months (95% CI: 33.0–NA); mOS: not reached). The subgroup with >5% of bone-marrow plasma cells and ≥3 plasmacytomas had the worst prognosis (mPFS: 19.3 months (95% CI: 13.4–28.8), *p* < 0.001; mOS: 27.9 months (95% CI: 19.3–67.8), *p* < 0.001). Our results show association between tumor burden and prognosis of NDMM patients with plasmacytomas. In the case of PS plasmacytomas, NDMM patients with low BM PC infiltration have an excellent prognosis.

## 1. Introduction

Multiple myeloma (MM) is the second-most-common hematologic malignancy; in Europe, the average incidence is 5/100,000 [1,2]. In the last decade, MM has slowly become a chronic disease due to novel agents such as proteasome inhibitors (PI), immunomodulatory drugs (IMIDs), and monoclonal antibodies [3,4,5,6].

MM is characterized by infiltration of the bone marrow (BM) by malignant plasma cells (PCs). In some cases, these PCs migrate out of the BM creating two types of plasmacytomas: paraskeletal lesions (tumors arising directly from the bone lesion; PS plasmacytomas) or extramedullary lesions (tumors infiltrating soft tissues; EMD plasmacytomas) possibly due to changes in adhesion molecules as well as rising independence of the BM [7,8,9]. Historically, plasmacytomas in MM were considered a high-risk feature [10]. In later analyses, EMD subsets of patients were shown to have the worst prognosis, while PS plasmacytomas had somehow better prognosis [11,12,13].

These plasmacytomas can be found in both newly diagnosed MM (NDMM) patients (2.4–11.5% of cases) [14,15,16] and in relapsed/refractory MM (RRMM) patients (3.4–27.4% of cases) [12,16,17,18]. The range of plasmacytoma incidence is predominantly caused by the different sensitivities of the imaging methods used [12,13,14,15,16,17,18], while historical methods, such as skeletal survey, have only a limited sensitivity for detection of PS or EMD plasmacytomas [7,8,9].

Although the appearance of plasmacytomas is generally associated with impaired prognosis, there are some treatment results of NDMM with plasmacytomas that are comparable to those of NDMM patients with strictly intra-medullar disease [14,19,20]. However, as we have previously described, development of plasmacytomas in RRMM patients is associated with extremely poor prognosis [12,18]. We believe that aggressivity of plasmacytomas in RRMM patients is likely due to disease-related factors. Molecular mechanisms of plasmacytoma development and expansion are still unclear, but especially in plasmacytomas of RRMM patients, mutations associated with poor prognosis (*TP53*, *K-RAS*, *N-RAS*, *RB1,* etc.), were often found. Similarly, the lack of adhesion molecules was found on PCs in plasmacytomas [7,8,9]. We have previously described NDMM patients with early progression with plasmacytomas. These patients had an aggressive disease course from the disease onset, together with gain (1q21) [18].

In this study, we evaluated clinical and laboratory data of one of the largest cohorts of NDMM patients with plasmacytomas to analyze their outcomes in real-life clinical practice conditions and to identify possible risk-groups.

## 2. Materials and Methods

### 2.1. Patient’ Selection

This multicentric real-life retrospective study was carried out in major hematologic centers in the Czech Republic between 2004 and 2021. For the data search of NDMM with plasmacytomas, the Registry of Monoclonal Gammopathies (RMG) of the Czech Myeloma Group was used. In total, 7123 NDMM patients fulfilling International Myeloma Working Group (IMWG) diagnostic criteria for MM were evaluated. We excluded all patients treated only with conventional chemotherapy or diagnosed only by skeletal survey; 523 NDMM patients with PS plasmacytomas, 196 NDMM patients with EMD plasmacytomas, and 2440 reference NDMM patients with clear absence of plasmacytoma (proven by high-sensitivity imaging methods) were identified. Of the enrolled patients with BM PCs <10% fulfilled MM diagnostic criteria with plasmacytoma/bone lesion tissue histology together with CRAB (i.e., osteolytic lesions, hypercalcemia etc.), or myeloma-defining events according to IMWG criteria. Patients with other plasma-cell dyscrasias (i.e., solitary plasmacytoma or solitary plasmacytoma with minimal marrow involvement) were not enrolled into the study. All participants provided written informed consent approved by institutional ethics boards in accordance with the latest Helsinki declaration.

### 2.2. Imaging Methods

The entire study cohort was evaluated for the presence of plasmacytomas by modern imaging methods—computed tomography (CT), focused/whole body magnetic resonance imaging (MRI/WB-MRI), or positron-emission tomography/computed tomography (PET/CT) [21]. Multiple diagnostic methods were performed on patients when clinically needed. If there was a clinical need and when safe for the patient, plasmacytomas were confirmed by surgical sampling.

### 2.3. Bone-Marrow Assessment

Bone-marrow samples were evaluated at the time of NDMM diagnosis. The number of BM PCs was evaluated by cytology, the clonality of BM PCs was evaluated by flow-cytometry, and interphase fluorescent in-situ hybridization (I-FISH) analyses of commonly found aberrations was performed on separated PCs as previously described [22].

### 2.4. Response Assessment and Survival Intervals

Treatment response was assessed according to the current International Myeloma Working Group (IMWG) criteria [23]. Survival intervals (progression-free survival, PFS and overall survival, OS) were assessed from the NDMM diagnosis.

### 2.5. Statistics

Data were described by absolute and relative frequencies of categorical variables and median with 5th–95th percentile range for quantitative variables. Fisher’s exact test was used to evaluate the association of selected features. The differences in survival (OS and PFS) among individual patient groups were assessed by the Kaplan–Meier method, and the statistical significance of differences in survival was evaluated using the log-rank test. The univariable Cox proportional-hazards model was used to quantify the effect of individual clinical features on the survival measures. The independence of selected features as prognostic survival factors was tested in the multivariable Cox proportional-hazards model in the context of R-ISS (Revised International Staging System). Statistical significance of hazard ratios (HR) was assessed by means of the Wald test. The cut-off for BM PCs was defined as the value where multivariable Cox regression adjusted to ISS showed highest HR and significance for OS and PFS, and the numbers of patients in the resulting groups was still sufficient. All statistical tests were performed at a significance level of α = 0.05 (all tests two-sided). Analysis was performed in the SPSS software (IBM Corp. Released 2017. IBM SPSS Statistics for Windows, Version 25.0.0.1 Armonk, NY, USA: IBM Corp.) and software R version 4.0.1. (www.r-project.org) [24].

## 3. Results

### 3.1. Clinical Characteristics of Patients

Altogether, 523 NDMM patients with PS plasmacytomas, 196 NDMM patients with EMD plasmacytomas, and 2440 reference NDMM patients were included into this study.

Median follow-up from diagnosis was 25.5 months (range 0.8–115.3 months) in the PS subgroup, 20.8 months (range 1.1–95.0 months) in the EMD subgroup, and 31.2 months (range 1.2–108.1 months) in reference patients. Clinical characteristics are summarized in Supplementary Table S1.

NDMM patients with PS and EMD plasmacytomas more frequently had lower BM PC infiltration (<10%) than reference patients (43.8% vs. 47.6% vs. 22.2%; *p* < 0.001). Correspondingly, these patients more frequently had a lower ratio of clonal BM PCs (<95% from all BM PCs) when compared to reference patients (32.2% vs. 38.7% vs 19.3%; *p* < 0.001).

In the PS subgroup, plasmacytomas were found by CT in 72.2% (380/523) of patients, by WB-MRI in 7.8% (41/523) of patients, by focused MRI in 38.4% (201/523) of patients, and by PET/CT in 31.0% (162/523) of patients. In 65.6% (343/523) of cases, only one plasmacytoma was found; in 9.8% (51/523) of patients two plasmacytomas were found, and in 12.6% (66/523), three and more plasmacytomas were found. In 12.0% (63/523) of patients, plasmacytoma count was missing.

In the EMD subgroup, plasmacytomas were found by CT in 64.3% (126/196) of patients, by WB-MRI in 12.7% (25/196) of patients, by focused MRI in 16.8% (33/196) of patients, and by PET/CT in 32.1% (63/196) of patients. In 68.8% (135/196) of cases, only one plasmacytoma was found; in 11.7% (23/196) of patients two plasmacytomas were found, and in 18.4% (36/196), three and more plasmacytomas were found. In 0.1% (2/196) of patients, plasmacytoma count was missing.

We also analyzed i-FISH data of all three subgroups of patients. Statistically significant differences between these groups of patients were not found.

### 3.2. Treatment of PS and EMD Subgroups of Patients after NDMM Diagnosis

In the PS subgroup, 82.6% (432/523) of patients were treated with PI, 57.2% (299/523) with IMIDs, 2.7% (14/523) with anti-CD38 monoclonal antibodies (daratumumab or isatuximab), and 35.6% (186/523) underwent high-dose chemotherapy followed by ASCT. Radiotherapy of plasmacytomas was administered in 31.7% (166/523) of these patients.

Similarly, in the EMD subgroup, 84.7% (166/196) of patients were treated with PI, 55.1% (108/196) with IMIDs, 2.0% (4/196) with anti-CD38 monoclonal antibodies (daratumumab or isatuximab), and 33.7% (66/196) underwent high-dose chemotherapy followed by ASCT. Radiotherapy of plasmacytomas was administered in 35.2% (69/196) of these patients.

Median PFS in the PS subgroup of patients was 25.8 months (95% CI: 22.7–28.6) which was significantly longer than in the EMD subgroup (17.9 months (95% CI: 15.0–22.3), *p* = 0.033), and longer than for the reference patients; however, statistical significance was not reached (23.3 months (95% CI: 22.5–24.8), *p* = 0.220).

Median OS in the PS subgroup was longer than for the EMD subgroup or reference patients but did not reach statistical significance (59.4 months (95% CI: 48.1–73.3) vs. 43.8 months (95% CI: 34.9–61.5) vs. 55.0 months (95% CI: 51.6–58.9), *p* = 0.229).

**Figure 1:**
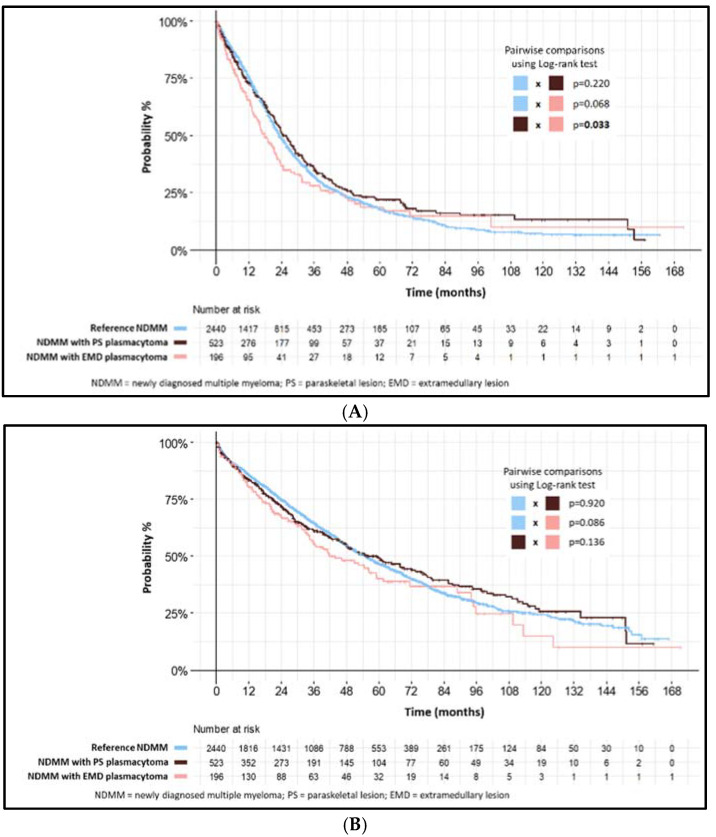
(**A**) PFS from 1st line—NDMM with plasmacytomas by type versus reference NDMM. (**B**) OS from 1st line—NDMM with plasmacytomas by type versus reference NDMM.

Treatment modalities and results including survival intervals are summarized in Figure 1 and Supplementary Table S2.

### 3.3. High-Risk Features in PS Subgroup of Patients

By univariable analysis, we found high R-ISS stage (R-ISS III) (HR 2.13 (95% CI: 1.24–3.66), *p* = 0.006), BM PC infiltration >5% (HR 2.38 (95 % CI: 1.67–3.39), *p* < 0.001), a higher ratio of clonal BM PCs (≥95% from all BM PCs) (HR 1.59 (95% CI: 1.08–2.32), *p* = 0.018), and higher LDH levels (>300 IU/L) (HR 2.50 (95 % CI: 1.76–3.56), *p* < 0.001) as statistically significant risk factors for inferior PFS in these patients. All these prognostic factors were also associated with inferior OS. Negative prognostic impact of BM PC infiltration >5% was more pronounced, when combined with higher PS plasmacytoma count (>5% BM PCs and ≥3 plasmacytomas) (PFS: HR 3.17 (95% CI: 1.93–5.20), *p* < 0.001; OS: HR 4.86 (95% CI: 2.69–8.80), *p* < 0.001).

According to i-FISH analysis, gain(1q21) was found to be a risk factor for inferior PFS (HR 1.67 (95 % CI: 1.20–2.33), *p* = 0.003). Moreover, t(4;14) (HR 1.87 (95 % CI: 1.14–3.05), *p* = 0.013), and del(17p13) (HR 1.74 (95 % CI: 1.09–2.78), *p* = 0.020) were found as risk factors for inferior PFS. Del(17p13) and t(4;14) were also associated with inferior OS. Other cytogenetic aberrations were not associated with adverse prognosis. Results of univariable analysis of risk factors in these patients are shown in Table 1.

By multivariable analysis adjusted for R-ISS stage, presence of PS plasmacytomas was not found as a risk factor for inferior PFS (HR 1.10 (95 % CI: 0.90–1.36), *p* = 0.343) and OS (HR 1.10 (95 % CI: 0.87–1.39), *p* = 0.431).

### 3.4. High-Risk Features in EMD Subgroup of Patients

By univariable analysis, we found high R-ISS stage (R-ISS III) (HR 5.03 (95% CI: 2.21–11.48), *p* < 0.001), ≥3 EMD plasmacytomas (HR 1.88 (95 % CI: 1.18–2.98), *p* = 0.008), and higher LDH levels (>300 IU/L) (HR 1.88 (95 % CI: 1.03–3.43), *p* = 0.041) as statistically significant risk factors for inferior PFS in these patients. All these prognostic factors were also associated with inferior OS. Interestingly, BM PC infiltration (>5%) or ratio of clonal BM PCs (>95%) had no prognostic impact.

According to i-FISH analysis, gain(1q21) (HR 1.82 (95 % CI: 1.11–2.99), *p* = 0.019) and del(17p13) (HR 2.96 (95 % CI: 1.46–6.00), *p* = 0.003) were found to be risk factors for inferior PFS as well as inferior OS. Results of univariable analysis of risk factors in these patients are summarized in Table 2.

By multivariable analysis adjusted for R-ISS, presence of EMD plasmacytomas was found as a risk factor for inferior PFS (HR 1.70 (95 % CI: 1.29–2.26), *p* < 0.001) and OS (HR 1.38 (95 % CI: 1.01–1.90), *p* = 0.046).

### 3.5. Heterogeneity in PS and EMD Subgroups of Patients

In addition to generally accepted prognostic markers, such as the ISS and R-ISS stage, we found intra- and/or extramedullary tumor burden to be an important prognostic indicator of both subgroups of plasmacytomas.

In the PS subgroup, we found <5% of BM PCs (regardless of plasmacytoma count) to have the best prognosis (mPFS: 58.3 months (95% CI: 33.0–NA), *p* < 0.001; mOS: not reached). These patients had more frequent low ISS stage (ISS I), low monoclonal immunoglobulin secretory activity, lower ratio of clonal BM PCs (<95% from all BM PCs), intact IgH gene, and relatively low plasmacytoma count.

**Figure 2:**
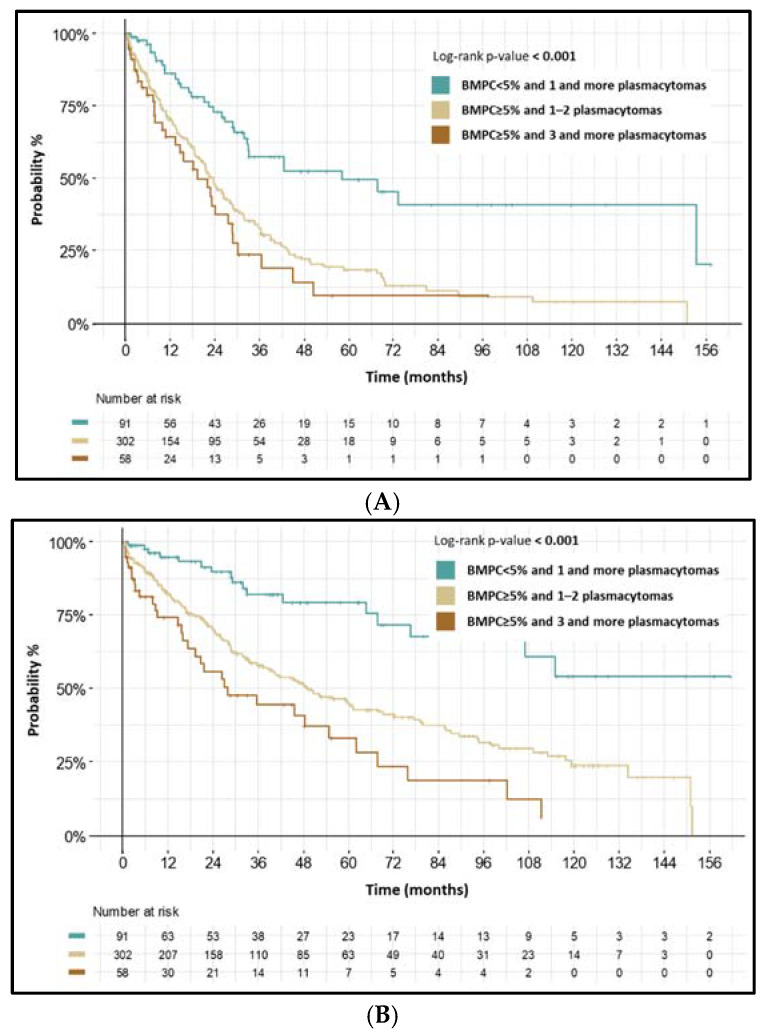
(**A**) PFS from 1st line—by tumor burden in NDMM patients with PS plasmacytomas. (**B**) OS from 1st line—by tumor burden in NDMM patients with PS plasmacytomas.

On the other hand, >5% of BM PCs together with 1–2 plasmacytomas had inferior prognosis (mPFS: 23.7 months, (95% CI: 20.1–27.2) *p* < 0.001; mOS: 49.1 months (95% CI: 39.8–68.0), *p* < 0.001); >5% of BM PCs together with ≥3 plasmacytomas had the worst prognosis (mPFS: 19.3 months (95% CI: 13.4–28.8), *p* <0.001; mOS: 27.9 months (95% CI: 19.3–67.8), *p* < 0.001). In these patients, high ISS (ISS III), high monoclonal immunoglobulin secretory activity, higher ratio of clonal BM PCs (≥95% from all BM PCs), higher count of bone osteolytic lesion (≥3 lesions), and higher frequency of IgH translocations were present. Characteristics and survival intervals of these three cohorts of PS subgroup are shown in Figure 2 and Table 3.

**Figure 3:**
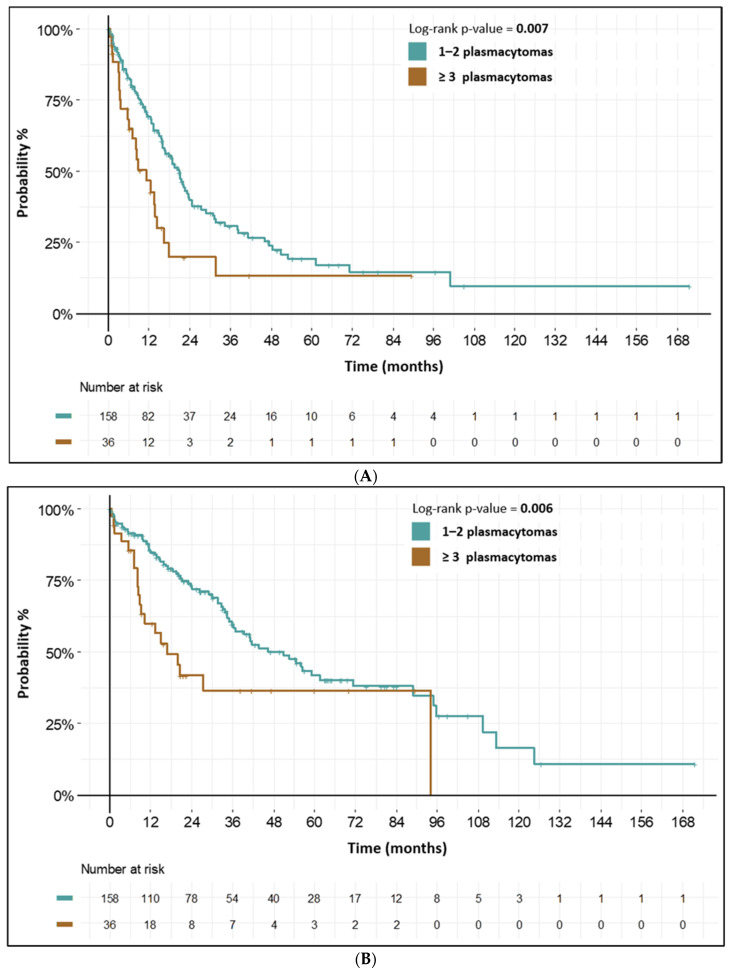
(**A**) PFS from 1st line—by plasmacytoma count in NDMM patients with EMD plasmacytomas. (**B**) OS from 1st line—by plasmacytoma count in NDMM patients with EMD plasmacytomas.

In the EMD subgroup with 1–2 EMD plasmacytomas, median PFS was 20.9 months (95% CI: 16.2–24.6) and median OS was 50.9 months (95% CI: 36.3–89.0). If ≥3 plasmacytomas were present, prognosis was significantly worse (median PFS 11.1 months (95% CI: 7.0–16.3), and median OS 16.9 months (95% CI: 9.1–NA)). Interestingly, there was no significant differences in ISS or R-ISS stage, BM PCs count, osteolytic lesions, cytogenetics, etc., between these cohorts of patients. Characteristics and survival intervals in the EMD subgroups of patients are shown in Figure 3 and Table 4.

## 4. Discussion

In the last decade, MM has gradually become a more manageable disease. Modern induction-treatment protocols lead to deeper responses and longer remissions in most NDMM patients [25,26,27,28]. Moreover, even treatment results of NDMM patients with plasmacytomas have improved when compared to historical data [13,14,19,20,29].

In this work, we analyzed NDMM patients with both paraskeletal and extramedullary plasmacytomas. As treatment regimens based on conventional chemotherapy are more than two decades obsolete in NDMM treatment [30], we censored historical patients treated with this approach from our analysis, with the aim of understanding the prognostic impact of plasmacytomas found in NDMM patients in real-life treatment scenarios. Similarly, as discussed above, we censored NDMM patients evaluated without high-sensitive imaging methods, such as CT, MRI, WB-MRI, or PET/CT.

In light of modern real-life treatment, we found NDMM patients with PS and EMD plasmacytomas to have comparable initial treatment results to those of NDMM patients without plasmacytomas (median PFS: 25.8 and 17.9 vs. 23.3 months; *p* = NS). Similarly to our analysis, in a study of NDMM with mostly PS plasmacytomas, predominantly IMID-based regimens lead to treatment results comparable to NDMM patients without plasmacytomas (median PFS: 25.3 months vs. 25.2 months; *p* = 0.46) [14]. In another analysis of NDMM patients treated with bortezomib-based induction, patients with single PS plasmacytoma had comparable treatment results to NDMM patients without any plasmacytomas (median PFS: 34.6 months vs. 38.1 months; *p* = 0.662). These results were improved with ASCT (median PFS: 46.0 months vs. 15.3 months; *p* = 0.073), but only 16% of patients with plasmacytomas in this study underwent ASCT [20]. Tandem ASCT did not show significant benefit in NDMM patients with plasmacytomas when compared to single ASCT [19] but may somehow have improved the inferior outcome of NDMM patients with plasmacytomas with high-risk cytogenetic aberrations [31]. Taken together, real-life induction protocols containing PIs, IMIDs and, in eligible patients, high-dose chemotherapy followed by ASCT, could change the outcome of NDMM patients with PS plasmacytomas.

Unfortunately, even our real-life dataset did not have many patients treated with anti-CD38 antibodies. While there is a lack of data about activity of anti-CD38 antibodies in the NDMM patients with plasmacytomas, data from RRMM patients with plasmacytomas are not promising [19,32].

In recently published papers, heterogeneity of MM patients with plasmacytomas is evident [7,8]. Moreover, this heterogeneity is strongly reflected in patients’ prognosis. It is obvious that the clinical course of NDMM patients with plasmacytomas and RRMM patients with plasmacytomas is dramatically different [12,14,18,19].

Further, there is a difference in clinical characteristics and prognosis between patients with EMD and PS plasmacytomas [7,8]. In accord with recent papers [7,8,33], our clinical and research groups have long considered PS and EMD plasmacytomas as very different entities in both NDMM and RRMM patients. Thus, we have studied them separately [9,12,18,34]. However, other groups describe all plasmacytomas outside of BM environment as one unit often making comparisons impossible [13,28,35].

The robust size of our dataset provides deeper insight, surprisingly revealing a significant heterogeneity even within subgroups of patients with PS or EMD plasmacytomas. This heterogeneity was reflected in far different prognoses.

In our study, the subgroup with PS plasmacytomas with very low BM PC (<5%) infiltration had surprisingly good prognosis; we found significantly more patients with clinical features associated with better prognosis, such as low ISS stage and lower proportion of clonal PCs in BM, called MGUS-like phenotype. MGUS-like phenotype is associated with an indolent clinical course and long survival [36]. in addition to low intramedullary tumor burden, plasmacytoma count was also low.

On the other hand, patients with higher BM PC infiltration together with increasing plasmacytoma count had worse prognosis. More patients with unfavorable characteristics were present, together with a higher frequency of numerous osteolytic lesions and higher paraprotein secretory activity. Our findings are supported by recent analyses, showing the negative prognostic impact of BM infiltration (cut-off >30% BM PCs) together with multiple plasmacytomas [20]. Similarly to other analyses, multiple PS plasmacytomas were also connected with inferior prognosis [11,14].

The most important result of our analysis was the prognostic impact of EMD plasmacytomas in NDMM patients. Generally, the presence of EMD plasmacytoma is a strong negative prognostic factor in MM patients, more pronounced in RRMM patients [11,12,13,16,18,19,35]. Interestingly, beside long-known prognostic indicators, such as ISS and R-ISS stage, we found EMD plasmacytoma count to be a strong biomarker of worse prognosis. The subgroup of our patients with numerous EMD plasmacytomas had dismal prognosis, with median overall survival near 17 months. On the other hand, we showed that in NDMM patients with a low number (1–2) of EMD plasmacytomas, treatment results were somehow comparable with NDMM patient without plasmacytoma presence (median PFS: 20.9 months vs. 23.3 months, median OS: 50.9 months vs. 55.0 months). Our results highlight the importance of high-sensitive imaging methods especially in patients with EMD plasmacytomas. According to the high prognostic impact of EMD plasmacytoma count, we recommend whole-body methods, such as PET/CT or WB-MRI in the standard diagnostic workup of NDMM patients with plasmacytomas [21].

These findings are in strict contrast with the dismal effect of EMD presence in RRMM patients [12,18,19] but, to the best of our knowledge, we have described the largest so-far published cohort of NDMM patients with EMD plasmacytomas detected by modern diagnostic methods and treated in real-life scenarios. Our findings are supported by another analysis of transplant-eligible NDMM patients where EMD patients with localized plasmacytoma involvement had a comparable 3-year PFS to that of NDMM patients without plasmacytomas (HR 1.03 (95% CI: 0.66-1.62; *p* = 0.88)), but patients with disseminated EMD plasmacytomas had worse prognosis (3-year PSF: HR 3.40 (95% CI: 1.74-6.61; *p* < 0.001) [11].

We found well-known high-risk cytogenetic aberrations del(17p) a t(4;14) retaining their negative prognostic impact predominantly in NDMM patients with PS plasmacytomas, as described in other studies [37,38]. Moreover, we found a negative prognostic impact of gain(1q21) in NDMM patients with EMD plasmacytomas. This finding is interesting, as we have previously published the higher risk of future plasmacytoma development in NDMM patients with gain(1q21) [18]. Similarly, analyses of a small groups of patients described higher incidence of gain(1q21) in NDMM patients with numerous EMD plasmacytomas [39,40]. Unfortunately, our results are based on a low number of evaluated samples. Another limitation was the absence of plasmacytoma tissue cytogenetics analysis in our work. Routine evaluation of plasmacytoma tissue can be complicated, mostly due to possible risk for patients from surgical sampling (i.e., paraspinal plasmacytomas and EMD in parenchymatous organs or the CNS).

## 5. Conclusions

Taken together, there is emerging evidence of the importance of the distinction between EMD and PS plasmacytomas in NDMM patients. Moreover, within these two entities, we found significant clinical heterogeneity, based on intra- and extramedullary tumor burden. These easy-to-assess biomarkers might reflect far different disease biology. Patients with PS plasmacytomas and low BM PC infiltration predominantly harbor low-risk features and have surprisingly good prognosis. In contrast, patients with higher BM PC infiltration and numerous PS plasmacytomas have poor prognosis. Prognosis of NDMM patients with EMD plasmacytomas is highly dependent on extramedullary burden. Patients with 1–2 EMD plasmacytomas had surprisingly comparable outcomes to NDMM patients without plasmacytoma. On the other hand, those with numerous EMD plasmacytomas had a dismal prognosis, resembling aggressivity of EMD plasmacytomas in RRMM patients.

## Figures and Tables

**Table 1:** Association of clinical features with OS and PFS in NDMM patients with PS plasmacytomas.

	Univariable Analysis			
	Overall Survival (OS)		Progression-Free Survival (PFS)	
	HR (95% CI) ^1^	*p*	HR (95% CI) ^1^	*p*
ISS				
Stage I	–	–	–	–
Stage II	2.01 (1.46–2.75)	<0.001	1.87 (1.41–2.48)	<0.001
Stage III	2.24 (1.60–3.13)	<0.001	2.30 (1.71–3.10)	<0.001
R-ISS				
Stage I	–	–	–	–
Stage II	3.00 (1.40–6.42)	0.005	1.73 (1.04–2.88)	0.035
Stage III	4.78 (2.20–10.38)	<0.001	2.13 (1.24–3.66)	0.006
Serum M-protein level (g/dL)				
≤2	–	–	–	–
>2	1.20 (0.92–1.55)	0.180	1.25 (0.98–1.58)	0.068
BM PCs %				
<5%	–	–	–	–
≥5%	3.12 (1.95–5.00)	<0.001	2.38 (1.67–3.39)	<0.001
Plasmacytoma count				
1–2 plasmacytomas	–	–	–	–
≥3 plasmacytomas	1.75 (1.20–2.55)	0.003	1.39 (0.97–1.99)	0.073
Tumor burden				
BM PCs < 5% and 1 and more plasmacytoma	–	–	–	–
BM PCs ≥ 5% and 1–2 plasmacytomas	3.01 (1.82–4.97)	<0.001	2.47 (1.68–3.62)	<0.001
BM PC ≥ 5% and 3 and more plasmacytomas	4.86 (2.69–8.80)	<0.001	3.17 (1.93–5.20)	<0.001
Clonal PCs from all BM PC (%)				
<95%	–	–	–	–
≥95%	2.01 (1.20–3.36)	0.008	1.59 (1.08–2.32)	0.018
Osteolytic lesions				
negative	–	–	–	–
1 lesion	1.10 (0.15–8.21)	0.925	0.52 (0.12–2.21)	0.375
2 lesions	1.33 (0.18–9.98)	0.779	0.90 (0.21–3.80)	0.882
≥3 lesions	1.36 (0.19–9.73)	0.758	0.89 (0.22–3.57)	0.866
Accelerated osteoporosis	0.54 (0.03–8.62)	0.662	0.57 (0.08–4.02)	0.569
LDH (IU/L)				
>300	2.52 (1.74–3.67)	<0.001	2.50 (1.76–3.56)	<0.001
IGH disruption				
Positive	1.18 (0.81–1.71)	0.385	1.16 (0.84–1.61)	0.378
t(11;14)				
Positive	1.39 (0.79–2.45)	0.258	1.03 (0.59–1.81)	0.913
t(4;14)				
Positive	2.37 (1.41–3.98)	0.001	1.87 (1.14–3.05)	0.013
Del(13)(q14)/monosomy 13				
Positive	1.44 (0.99–2.10)	0.059	1.12 (0.80–1.55)	0.516
Gain(1q21)				
Positive	1.32 (0.90–1.93)	0.157	1.67 (1.20–2.33)	0.003
Del(17p13)				
Positive	2.17 (1.32–3.56)	0.002	1.74 (1.09–2.78)	0.020
Hyperdiploidy				
Positive	0.64 (0.41–0.99)	0.045	0.79 (0.54–1.16)	0.227

^1^ Hazard ratio (HR) from univariable Cox’s proportional hazard model. Abbreviations: ISS, International Staging System; R-ISS, Revised-ISS; BM PCs, bone-marrow plasma cells; LDH, lactate dehydrogenase; IGH, immunoglobulin heavy chain.

**Table 2:** Association of clinical features with OS and PFS in NDMM patients with EMD plasmacytomas.

	Univariable Analysis			
	Overall Survival (OS)		Progression-Free Survival (PFS)	
	HR (95% CI) ^1^	*p*	HR (95% CI) ^1^	*p*
ISS				
Stage I	–	–	–	–
Stage II	1.49 (0.87–2.56)	0.151	1.48 (0.93–2.37)	0.102
Stage III	3.21 (1.94–5.31)	<0.001	2.49 (1.60–3.89)	<0.001
R-ISS				
Stage I	–	–	–	–
Stage II	1.77 (0.63–4.93)	0.276	2.07 (0.93–4.63)	0.075
Stage III	5.64 (2.08–15.26)	0.001	5.03 (2.21–11.48)	<0.001
Serum M-protein level (g/dL)				
>2	1.13 (0.75–1.71)	0.565	1.34 (0.93–1.94)	0.122
BM PCs %				
<5%	–	–	–	–
≥5%	1.30 (0.76–2.20)	0.338	1.56 (0.97–2.52)	0.066
Plasmacytoma count				
1–2 lesions	–	–	–	–
≥3 lesions	2.00 (1.21–3.30)	0.007	1.88 (1.18–2.98)	0.008
Clonal PCs from all BM PC (%)				
≥95%	1.36 (0.72–2.59)	0.346	1.41 (0.83–2.39)	0.210
Osteolytic lesions				
Negative	–	–	–	–
1 lesion	0.33 (0.11–1.03)	0.055	0.56 (0.16–2.04)	0.382
2 lesions	0.26 (0.07–0.90)	0.033	0.45 (0.12–1.70)	0.236
≥3 lesions	0.49 (0.20–1.21)	0.122	0.65 (0.20–2.06)	0.461
Accelerated osteoporosis	0.39 (0.09–1.62)	0.194	0.52 (0.12–2.19)	0.372
LDH (IU/L)				
> 300	2.29 (1.20–4.38)	0.012	1.88 (1.03–3.43)	0.041
IGH disruption				
Positive	1.19 (0.67–2.12)	0.557	1.14 (0.69–1.89)	0.612
t(11;14)				
Positive	1.01 (0.31–3.33)	0.988	0.57 (0.14–2.37)	0.441
t(4;14)				
Positive	0.77 (0.33–1.78)	0.542	1.55 (0.80–2.99)	0.191
Del(13)(q14)/monosomy 13				
Positive	1.06 (0.59–1.90)	0.851	1.53 (0.94–2.49)	0.087
Gain(1q21)				
Positive	1.86 (1.06–3.27)	0.031	1.82 (1.11–2.99)	0.019
Del(17p13)				
Positive	2.62 (1.21–5.67)	0.014	2.96 (1.46–6.00)	0.003
Hyperdiploidy				
Positive	1.06 (0.55–2.02)	0.867	1.18 (0.65–2.15)	0.593

^1^ Hazard ratio (HR) from univariable Cox’s proportional hazard model. Abbreviations: ISS, International Staging System; R-ISS, Revised-ISS; BM PCs, bone marrow plasma cells; LDH, lactate dehydrogenase; IGH, immunoglobulin heavy chain.

**Table 3:** Descriptive characteristics according to tumor burden in NDMM patients with PS plasmacytomas.

Characteristics ^1^	BM PCs < 5% and 1 or More Plasmacytomas (*n* = 91)	BM PCs ≥ 5% and 1–2 Plasmacytomas (*n* = 302)	BM PCs ≥ 5% and ≥3 Plasmacytomas (*n* = 58)	*p*-Value ^2^
ISS	*n* = 91	*n* = 298	*n* = 58	
Stage I	63 (69.2%)	113 (37.9%)	17 (29.3%)	<0.001
Stage II	16 (17.6%)	103 (34.6%)	17 (29.3%)	
Stage III	12 (13.2%)	82 (27.5%)	24 (41.4%)	
R-ISS	*n* = 18	*n* = 120	*n* = 38	
Stage I	7 (38.9%)	25 (20.8%)	4 (10.5%)	0.195
Stage II	7 (38.9%)	63 (52.5%)	21 (55.3%)	
Stage III	4 (22.2%)	32 (26.7%)	13 (34.2%)	
Serum M-protein level (g/dL)	*n* = 91	*n* = 302	*n* = 58	
≤2	66 (72.5%)	145 (48.0%)	26 (44.8%)	<0.001
>2	25 (27.5%)	157 (52.0%)	32 (55.2%)	
Plasmacytoma count	*n* = 91	*n* = 302	*n* = 58	
1 plasmacytoma	73 (80.2%)	263 (87.1%)	-	<0.001
2 plasmacytomas	12 (13.2%)	39 (12.9%)	-	
3 plasmacytomas	1 (1.1%)	-	16 (27.6%)	
>3 plasmacytomas	5 (5.5%)	-	42 (72.4%)	
Clonal PCs from all BM PC (%)	*n* = 57	*n* = 159	*n* = 25	
<95%	38 (66.7%)	37 (23.3%)	1 (4.0%)	<0.001
≥95%	19 (33.3%)	122 (76.7%)	24 (96.0%)	
Osteolytic lesions	*n* = 91	*n* = 302	*n* = 58	
Negative	0 (0.0%)	2 (0.7%)	0 (0.0%)	<0.001
1 lesion	17 (18.7%)	32 (10.6%)	0 (0.0%)	
2 lesions	3 (3.3%)	28 (9.3%)	0 (0.0%)	
≥3 lesions	70 (76.9%)	238 (78.8%)	58 (100.0%)	
Accelerated osteoporosis	1 (1.1%)	2 (0.7%)	0 (0.0%)	
LDH (IU/L)	*n* = 91	*n* = 296	*n* = 58	
≤300	84 (92.3%)	271 (91.6%)	53 (91.4%)	1.000
>300	7 (7.7%)	25 (8.4%)	5 (8.6%)	
IGH disruption	*n* = 24	*n* = 183	*n* = 37	
Negative	22 (91.7%)	106 (57.9%)	18 (48.6%)	0.001
Positive	2 (8.3%)	77 (42.1%)	19 (51.4%)	
t(11;14)	*n* = 23	*n* = 156	*n* = 32	
Negative	22 (95.7%)	135 (86.5%)	25 (78.1%)	0.195
Positive	1 (4.3%)	21 (13.5%)	7 (21.9%)	
t(4;14)	*n* = 25	*n* = 161	*n* = 34	
Negative	25 (100.0%)	145 (90.1%)	29 (85.3%)	0.119
Positive	0 (0.0%)	16 (9.9%)	5 (14.7%)	
Del(13)(q14)/monosomy 13	*n* = 24	*n* = 183	*n* = 36	
Negative	14 (58.3%)	100 (54.6%)	20 (55.6%)	0.975
Positive	10 (41.7%)	83 (45.4%)	16 (44.4%)	
Gain(1q21)	*n* = 24	*n* = 176	*n* = 38	
Negative	17 (70.8%)	111 (63.1%)	19 (50.0%)	0.203
Positive	7 (29.2%)	65 (36.9%)	19 (50.0%)	
Del(17p13)	*n* = 24	*n* = 165	*n* = 35	
Negative	24 (100.0%)	146 (88.5%)	30 (85.7%)	0.141
Positive	0 (0.0%)	19 (11.5%)	5 (14.3%)	
Hyperdiploidy	*n* = 20	*n* = 129	*n* = 36	
Negative	12 (60.0%)	75 (58.1%)	20 (55.6%)	0.945
Positive	8 (40.0%)	54 (41.9%)	16 (44.4%)	

^1^ Described by absolute and relative frequencies for categorical variables and median (5th–95th percentile) for continuous variables. ^2^ *p*-value of Fisher’s exact test for categorical variables or Kruskal–Wallis test for continuous variables. Abbreviations: ISS, International Staging System; R-ISS, Revised-ISS; BM PCs, bone marrow plasma cells; LDH, lactate dehydrogenase; IGH, immunoglobulin heavy chain.

**Table 4:** Descriptive characteristics according to plasmacytoma count in NDMM patients with EMD plasmacytomas.

Characteristics ^1^	1–2 EMD Plasmacytomas (*n* = 158)	≥3 EMD Plasmacytomas (*n* = 36)	*p*-Value ^2^
ISS	*n* = 156	*n* = 36	
Stage I	68 (43.6%)	11 (30.6%)	0.189
Stage II	43 (27.6%)	9 (25.0%)	
Stage III	45 (28.8%)	16 (44.4%)	
R-ISS	*n* = 65	*n* = 21	
Stage I	17 (26.2%)	3 (14.3%)	0.173
Stage II	26 (40.0%)	6 (28.6%)	
Stage III	22 (33.8%)	12 (57.1%)	
Serum M-protein level (g/dL)	*n* = 158	*n* = 36	
≤ 2	81 (51.3%)	18 (50.0%)	1.000
> 2	77 (48.7%)	18 (50.0%)	
Clonal PCs from all BM PCs (%)	*n* = 95	*n* = 15	
<95%	37 (38.9%)	6 (40.0%)	1.000
≥95%	58 (61.1%)	9 (60.0%)	
BM PCs %	*n* = 152	*n* = 33	
<5%	38 (25.0%)	9 (27.3%)	0.826
≥5%	114 (75.0%)	24 (72.7%)	
Osteolytic lesions	*n* = 156	*n* = 36	
Negative	6 (3.8%)	0 (0.0%)	0.115
1 lesion	18 (11.5%)	2 (5.6%)	
2 lesions	15 (9.6%)	0 (0.0%)	
≥3 lesions	110 (70.5%)	32 (88.9%)	
Accelerated osteoporosis	7 (4.5%)	2 (5.6%)	
LDH (IU/L)	*n* = 158	*n* = 36	
≤ 300	146 (92.4%)	31 (86.1%)	0.322
> 300	12 (7.6%)	5 (13.9%)	
IGH disruption	*n* = 88	*n* = 17	
Negative	58 (65.9%)	9 (52.9%)	0.409
Positive	30 (34.1%)	8 (47.1%)	
t(11;14)	*n* = 72	*n* = 14	
Negative	67 (93.1%)	12 (85.7%)	0.319
Positive	5 (6.9%)	2 (14.3%)	
t(4;14)	*n* = 86	*n* = 17	
Negative	76 (88.4%)	16 (94.1%)	0.686
Positive	10 (11.6%)	1 (5.9%)	
Del(13)(q14)/monosomy 13	*n* = 89	*n* = 17	
Negative	53 (59.6%)	11 (64.7%)	0.791
Positive	36 (40.4%)	6 (35.3%)	
Gain(1q21)	*n* = 88	*n* = 17	
Negative	51 (58.0%)	7 (41.2%)	0.287
Positive	37 (42.0%)	10 (58.8%)	
del(17p13)	*n* = 86	*n* = 15	
Negative	72 (83.7%)	11 (73.3%)	0.462
Positive	14 (16.3%)	4 (26.7%)	
Hyperdiploidy	*n* = 61	*n* = 14	
Negative	30 (49.2%)	5 (35.7%)	0.393
Positive	31 (50.8%)	9 (64.3%)	

^1^ Described by absolute and relative frequencies for categorical variables and median (5th–95th percentile) for continuous variables. ^2^ *p*-value of Fisher’s exact test for categorical variables or Kruskal–Wallis test for continuous variables. Abbreviations: ISS (International Staging System), R-ISS (Revised-ISS) BM PCs (bone marrow plasma cells), LDH (lactate dehydrogenase), IGH (immunoglobulin heavy chain).

## Data Availability

Data are available upon request.

## Supplementary Materials

The following supporting information can be downloaded at: https://www.mdpi.com/article/10.3390/biomedicines10102535/s1, Table S1. Descriptive characteristics of patients’ groups at NDMM diagnosis. Table S2. Treatment in first line of therapy.

## References

[1] Sant M., Allemani C., Tereanu C., De Angelis R., Capocaccia R., Visser O., Marcos-Gragera R., Maynadié M., Simonetti A., Lutz J.-M. (2010). Incidence of Hematologic Malignancies in Europe by Morphologic Subtype: Results of the HAEMACARE Project. Blood.

[2] Maluskova D., Svobodová I., Kucerova M., Brozova L., Muzik J., Jarkovský J., Hájek R., Maisnar V., Dusek L. (2017). Epidemiology of Multiple Myeloma in the Czech Republic. Klin. Onkol. Cas. Ceske Slov. Onkol. Spol..

[3] Cavo M., San-Miguel J., Usmani S.Z., Weisel K., Dimopoulos M.A., Avet-Loiseau H., Paiva B., Bahlis N.J., Plesner T., Hungria V. (2022). Prognostic Value of Minimal Residual Disease Negativity in Myeloma: Combined Analysis of POLLUX, CASTOR, ALCYONE, and MAIA. Blood.

[4] Rodriguez-Otero P., Paiva B., San-Miguel J.F. (2021). Roadmap to Cure Multiple Myeloma. Cancer Treat. Rev..

[5] Kumar S.K., Rajkumar V., Kyle R.A., van Duin M., Sonneveld P., Mateos M.-V., Gay F., Anderson K.C. (2017). Multiple Myeloma. Nat. Rev. Dis. Primer.

[6] Morgan G.J., Rasche L. (2017). Haematological Cancer: Where Are We Now with the Treatment of Multiple Myeloma?. Nat. Rev. Clin. Oncol..

[7] Bladé J., Beksac M., Caers J., Jurczyszyn A., von Lilienfeld-Toal M., Moreau P., Rasche L., Rosiñol L., Usmani S.Z., Zamagni E. (2022). Extramedullary Disease in Multiple Myeloma: A Systematic Literature Review. Blood Cancer J..

[8] Rosiñol L., Beksac M., Zamagni E., Van de Donk N.W.C.J., Anderson K.C., Badros A., Caers J., Cavo M., Dimopoulos M.-A., Dispenzieri A. (2021). Expert Review on Soft-Tissue Plasmacytomas in Multiple Myeloma: Definition, Disease Assessment and Treatment Considerations. Br. J. Haematol..

[9] Sevcikova S., Minarik J., Stork M., Jelinek T., Pour L., Hajek R. (2019). Extramedullary Disease in Multiple Myeloma–Controversies and Future Directions. Blood Rev..

[10] Durie B.G.M. (2006). The Role of Anatomic and Functional Staging in Myeloma: Description of Durie/Salmon plus Staging System. Eur. J. Cancer Oxf. Engl. 1990.

[11] Gagelmann N., Eikema D.-J., Iacobelli S., Koster L., Nahi H., Stoppa A.-M., Masszi T., Caillot D., Lenhoff S., Udvardy M. (2018). Impact of Extramedullary Disease in Patients with Newly Diagnosed Multiple Myeloma Undergoing Autologous Stem Cell Transplantation: A Study from the Chronic Malignancies Working Party of the EBMT. Haematologica.

[12] Pour L., Sevcikova S., Greslikova H., Kupska R., Majkova P., Zahradova L., Sandecka V., Adam Z., Krejci M., Kuglik P. (2014). Soft-Tissue Extramedullary Multiple Myeloma Prognosis Is Significantly Worse in Comparison to Bone-Related Extramedullary Relapse. Haematologica.

[13] Varettoni M., Corso A., Pica G., Mangiacavalli S., Pascutto C., Lazzarino M. (2010). Incidence, Presenting Features and Outcome of Extramedullary Disease in Multiple Myeloma: A Longitudinal Study on 1003 Consecutive Patients. Ann. Oncol. Off. J. Eur. Soc. Med. Oncol..

[14] Montefusco V., Gay F., Spada S., Paoli L.D., Raimondo F.D., Ribolla R., Musolino C., Patriarca F., Musto P., Galieni P. (2020). Outcome of Paraosseous Extra-Medullary Disease in Newly Diagnosed Multiple Myeloma Patients Treated with New Drugs. Haematologica.

[15] Short K.D., Rajkumar S.V., Larson D., Buadi F., Hayman S., Dispenzieri A., Gertz M., Kumar S., Mikhael J., Roy V. (2011). Incidence of Extramedullary Disease in Patients with Multiple Myeloma in the Era of Novel Therapy, and the Activity of Pomalidomide on Extramedullary Myeloma. Leukemia.

[16] Usmani S.Z., Heuck C., Mitchell A., Szymonifka J., Nair B., Hoering A., Alsayed Y., Waheed S., Haider S., Restrepo A. (2012). Extramedullary Disease Portends Poor Prognosis in Multiple Myeloma and Is Over-Represented in High-Risk Disease Even in the Era of Novel Agents. Haematologica.

[17] Varga C., Xie W., Laubach J., Ghobrial I.M., O’Donnell E.K., Weinstock M., Paba-Prada C., Warren D., Maglio M.E., Schlossman R. (2015). Development of Extramedullary Myeloma in the Era of Novel Agents: No Evidence of Increased Risk with Lenalidomide-Bortezomib Combinations. Br. J. Haematol..

[18] Stork M., Sevcikova S., Minarik J., Krhovska P., Radocha J., Pospisilova L., Brozova L., Jarkovsky J., Spicka I., Straub J. (2022). Identification of Patients at High Risk of Secondary Extramedullary Multiple Myeloma Development. Br. J. Haematol..

[19] Beksac M., Seval G.C., Kanellias N., Coriu D., Rosiñol L., Ozet G., Goranova-Marinova V., Unal A., Bila J., Ozsan H. (2020). A Real World Multicenter Retrospective Study on Extramedullary Disease from Balkan Myeloma Study Group and Barcelona University: Analysis of Parameters That Improve Outcome. Haematologica.

[20] He J., Yue X., He D., Zhao Y., Yang Y., Zheng G., Zhang E., Han X., Wu W., Yang L. (2021). Multiple Extramedullary-Bone Related and/or Extramedullary Extraosseous Are Independent Poor Prognostic Factors in Patients With Newly Diagnosed Multiple Myeloma. Front. Oncol..

[21] Zamagni E., Nanni C., Dozza L., Carlier T., Bailly C., Tacchetti P., Versari A., Chauvie S., Gallamini A., Gamberi B. (2021). Standardization of 18F-FDG-PET/CT According to Deauville Criteria for Metabolic Complete Response Definition in Newly Diagnosed Multiple Myeloma. J. Clin. Oncol. Off. J. Am. Soc. Clin. Oncol..

[22] Besse L., Sedlarikova L., Greslikova H., Kupska R., Almasi M., Penka M., Jelinek T., Pour L., Adam Z., Kuglik P. (2016). Cytogenetics in Multiple Myeloma Patients Progressing into Extramedullary Disease. Eur. J. Haematol..

[23] Rajkumar S.V., Dimopoulos M.A., Palumbo A., Blade J., Merlini G., Mateos M.-V., Kumar S., Hillengass J., Kastritis E., Richardson P. (2014). International Myeloma Working Group Updated Criteria for the Diagnosis of Multiple Myeloma. Lancet Oncol..

[24] R Core Team (2015). R: A Language and Environment for Statistical Computing.

[25] Mateos M.-V., Cavo M., Blade J., Dimopoulos M.A., Suzuki K., Jakubowiak A., Knop S., Doyen C., Lucio P., Nagy Z. (2020). Overall Survival with Daratumumab, Bortezomib, Melphalan, and Prednisone in Newly Diagnosed Multiple Myeloma (ALCYONE): A Randomised, Open-Label, Phase 3 Trial. Lancet Lond. Engl..

[26] Facon T., Kumar S., Plesner T., Orlowski R.Z., Moreau P., Bahlis N., Basu S., Nahi H., Hulin C., Quach H. (2019). Daratumumab plus Lenalidomide and Dexamethasone for Untreated Myeloma. N. Engl. J. Med..

[27] Yimer H., Melear J., Faber E., Bensinger W.I., Burke J.M., Narang M., Stevens D., Gunawardena S., Lutska Y., Qi K. (2019). Daratumumab, Bortezomib, Cyclophosphamide and Dexamethasone in Newly Diagnosed and Relapsed Multiple Myeloma: LYRA Study. Br. J. Haematol..

[28] Landgren O., Sonneveld P., Jakubowiak A., Mohty M., Iskander K.S., Mezzi K., Siegel D.S. (2019). Carfilzomib with Immunomodulatory Drugs for the Treatment of Newly Diagnosed Multiple Myeloma. Leukemia.

[29] Wu P., Davies F.E., Boyd K., Thomas K., Dines S., Saso R.M., Potter M.N., Ethell M.E., Shaw B.E., Morgan G.J. (2009). The Impact of Extramedullary Disease at Presentation on the Outcome of Myeloma. Leuk. Lymphoma.

[30] Attal M., Harousseau J.L., Stoppa A.M., Sotto J.J., Fuzibet J.G., Rossi J.F., Casassus P., Maisonneuve H., Facon T., Ifrah N. (1996). A Prospective, Randomized Trial of Autologous Bone Marrow Transplantation and Chemotherapy in Multiple Myeloma. Intergroupe Français Du Myélome. N. Engl. J. Med..

[31] Gagelmann N., Eikema D.-J., Koster L., Caillot D., Pioltelli P., Lleonart J.B., Reményi P., Blaise D., Schaap N., Trneny M. (2019). Tandem Autologous Stem Cell Transplantation Improves Outcomes in Newly Diagnosed Multiple Myeloma with Extramedullary Disease and High-Risk Cytogenetics: A Study from the Chronic Malignancies Working Party of the European Society for Blood and Marrow Transplantation. Biol. Blood Marrow Transplant. J. Am. Soc. Blood Marrow Transplant..

[32] Jelinek T., Sevcikova T., Zihala D., Popkova T., Kapustova V., Broskevicova L., Capkova L., Rihova L., Bezdekova R., Sevcikova S. (2022). Limited Efficacy of Daratumumab in Multiple Myeloma with Extramedullary Disease. Leukemia.

[33] Bansal R., Rakshit S., Kumar S. (2021). Extramedullary Disease in Multiple Myeloma. Blood Cancer J..

[34] Gregorova J., Vychytilova-Faltejskova P., Kramarova T., Knechtova Z., Almasi M., Stork M., Pour L., Kohoutek J., Sevcikova S. (2022). Proteomic Analysis of the Bone Marrow Microenvironment in Extramedullary Multiple Myeloma Patients. Neoplasma.

[35] Weinstock M., Aljawai Y., Morgan E.A., Laubach J., Gannon M., Roccaro A.M., Varga C., Mitsiades C.S., Paba-Prada C., Schlossman R. (2015). Incidence and Clinical Features of Extramedullary Multiple Myeloma in Patients Who Underwent Stem Cell Transplantation. Br. J. Haematol..

[36] Paiva B., Vídriales M.-B., Rosiñol L., Martínez-López J., Mateos M.-V., Ocio E.M., Montalbán M.-Á., Cordón L., Gutiérrez N.C., Corchete L. (2013). A Multiparameter Flow Cytometry Immunophenotypic Algorithm for the Identification of Newly Diagnosed Symptomatic Myeloma with an MGUS-like Signature and Long-Term Disease Control. Leukemia.

[37] Abdallah N., Rajkumar S.V., Greipp P., Kapoor P., Gertz M.A., Dispenzieri A., Baughn L.B., Lacy M.Q., Hayman S.R., Buadi F.K. (2020). Cytogenetic Abnormalities in Multiple Myeloma: Association with Disease Characteristics and Treatment Response. Blood Cancer J..

[38] Sonneveld P., Avet-Loiseau H., Lonial S., Usmani S., Siegel D., Anderson K.C., Chng W.-J., Moreau P., Attal M., Kyle R.A. (2016). Treatment of Multiple Myeloma with High-Risk Cytogenetics: A Consensus of the International Myeloma Working Group. Blood.

[39] Qu X., Chen L., Qiu H., Lu H., Wu H., Qiu H., Liu P., Guo R., Li J. (2015). Extramedullary Manifestation in Multiple Myeloma Bears High Incidence of Poor Cytogenetic Aberration and Novel Agents Resistance. BioMed Res. Int..

[40] Biran N., Malhotra J., Bagiella E., Cho H.J., Jagannath S., Chari A. (2014). Patients with Newly Diagnosed Multiple Myeloma and Chromosome 1 Amplification Have Poor Outcomes despite the Use of Novel Triplet Regimens. Am. J. Hematol..

