# Supplementary material for: Unexpected Heterogeneity of Newly Diagnosed Multiple Myeloma Patients with Plasmacytomas
Source: Biomedicines. 2022 Oct 11;10(10):2535. doi: 10.3390/biomedicines10102535 (PMC9599767; doi:10.3390/biomedicines10102535)
Supplement: Supplementary file 1 [file biomedicines-10-02535-s001.zip › biomedicines-1936855-supplementary.pdf]

Supplementary Table S1: Descriptive characteristics of patients` groups at NDMM diagnosis

Characteristics [1]	NDMM reference (N=2440)	NDMM with PS plasmacytomas (N=523)	NDMM with EMD plasmacytomas (N=196)	p-value[2]
**Sex**	n=2440	n=523	n=196	
Women	1148 (47.0%)	210 (40.2%)	76 (38.8%)	**0.002**
Men	1292 (53.0%)	313 (59.8%)	120 (61.2%)	
**Age at tr. initiation (years)**	n=2440	n=523	n=196	
≤ 65	1063 (43.6%)	271 (51.8%)	93 (47.4%)	
66–75	974 (39.9%)	173 (33.1%)	72 (36.7%)	**0.013**
>75	403 (16.5%)	79 (15.1%)	31 (15.8%)	
**ECOG PS**	n=2431	n=521	n=196	
0–1	1656 (68.1%)	315 (60.5%)	114 (58.2%)	
2	516 (21.2%)	134 (25.7%)	47 (24.0%)	**<0.001**
3–4	259 (10.7%)	72 (13.8%)	35 (17.9%)	
**ISS**	n=2398	n=518	n=194	
Stage 1	682 (28.4%)	213 (41.1%)	79 (40.7%)	
Stage 2	789 (32.9%)	163 (31.5%)	54 (27.8%)	**<0.001**
Stage 3	927 (38.7%)	142 (27.4%)	61 (31.4%)	
**R-ISS**	n=1007	n=205	n=87	
Stage 1	128 (12.7%)	39 (19.0%)	20 (23.0%)	
Stage 2	444 (44.1%)	102 (49.8%)	33 (37.9%)	**0.001**
Stage 3	435 (43.2%)	64 (31.2%)	34 (39.1%)	
**M-protein type**	n=2438	n=522	n=196	
IgG	1426 (58.5%)	279 (53.4%)	116 (59.2%)	
IgA	514 (21.1%)	115 (22.0%)	35 (17.9%)	
LC only	395 (16.2%)	106 (20.3%)	32 (16.3%)	0.147
Non-secretory	36 (1.5%)	8 (1.5%)	7 (3.6%)	
other	67 (2.7%)	14 (2.7%)	6 (3.1%)	
**Serum M-protein level (g/dL)**	n=2431	n=520	n=196	
≤ 2	1011 (41.6%)	276 (53.1%)	100 (51.0%)	**<0.001**
> 2	1420 (58.4%)	244 (46.9%)	96 (49.0%)	
**BMPCs %**	n=2360	n=511	n=187	
<10%	525 (22.2%)	224 (43.8%)	89 (47.6%)	**<0.001**
≥10%	1835 (77.8%)	287 (56.2%)	98 (52.4%)	
**Clonal PC (%) - flow cytometry**	n=1081	n=264	n=111	
<95%	209 (19.3%)	85 (32.2%)	43 (38.7%)	**<0.001**
≥95%	872 (80.7%)	179 (67.8%)	68 (61.3%)	
**LDH (IU/L)**	n=2374	n=515	n=196	
≤ 210	1541 (64.9%)	317 (61.6%)	126 (64.3%)	0.352
> 210	833 (35.1%)	198 (38.4%)	70 (35.7%)	

**Creatinine level (µmol/L)**	n=2436	n=522	n=196	
≤ 176	1955 (80.3%)	457 (87.5%)	171 (87.2%)	**<0.001**
> 176	481 (19.7%)	65 (12.5%)	25 (12.8%)	
**Calcium total level (mmol/L)**	n=2435	n=522	n=196	
≤ 2.65	2079 (85.4%)	452 (86.6%)	167 (85.2%)	0.763
> 2.65	356 (14.6%)	70 (13.4%)	29 (14.8%)	
**Hemoglobin level (g/dL)**	n=2438	n=522	n=196	
≥ 12	673 (27.6%)	220 (42.1%)	86 (43.9%)	**<0.001**
< 12	1765 (72.4%)	302 (57.9%)	110 (56.1%)	
**Thrombocyte count (10E9/L)**	n=2437	n=522	n=196	
≥ 175	1666 (68.4%)	417 (79.9%)	158 (80.6%)	**<0.001**
< 175	771 (31.6%)	105 (20.1%)	38 (19.4%)	
**IGH disruption**	n=1315	n=268	n=107	
negative	736 (56.0%)	160 (59.7%)	68 (63.6%)	0.203
positive	579 (44.0%)	108 (40.3%)	39 (36.4%)	
**t(11;14)**	n=1119	n=231	n=87	
negative	951 (85.0%)	199 (86.1%)	80 (92.0%)	0.196
positive	168 (15.0%)	32 (13.9%)	7 (8.0%)	
**t(4;14)**	n=1194	n=244	n=105	
negative	1035 (86.7%)	221 (90.6%)	93 (88.6%)	0.239
positive	159 (13.3%)	23 (9.4%)	12 (11.4%)	
**del(13)(q14)/monosomy 13**	n=1332	n=268	n=107	
negative	705 (52.9%)	143 (53.4%)	65 (60.7%)	0.301
positive	627 (47.1%)	125 (46.6%)	42 (39.3%)	
**gain(1q21)**	n=1310	n=262	n=107	
negative	762 (58.2%)	164 (62.6%)	59 (55.1%)	0.306
positive	548 (41.8%)	98 (37.4%)	48 (44.9%)	
**del(17p13)**	n=1235	n=246	n=102	
negative	1070 (86.6%)	216 (87.8%)	84 (82.4%)	0.388
positive	165 (13.4%)	30 (12.2%)	18 (17.6%)	
**Hyperdiploidy**	n=1115	n=212	n=76	
negative	615 (55.2%)	124 (58.5%)	35 (46.1%)	0.179
positive	500 (44.8%)	88 (41.5%)	41 (53.9%)	

[1] described by absolute and relative frequencies for categorical variables and median (5th–95th percentile) for continuous variables

2 p-value of Fisher's exact test for categorical variables or Kruskal-Wallis test for continuous variables

Supplementary Table S2: Treatment in 1st line of therapy

**Characteristics** [1]	**NDMM reference (N=2440)**	**NDMM with PS plasmacytoma (N=523)**	**NDMM with EMD plasmacytoma (N=196)**	**p-value**[2]
**Proteasome inhibitors**	n=2440	n=523	n=196	
no	568 (23.3%)	91 (17.4%)	30 (15.3%)	**0.001**
yes	1872 (76.7%)	432 (82.6%)	166 (84.7%)	
**Immunomodulatory drugs**	n=2440	n=523	n=196	
no	1033 (42.3%)	224 (42.8%)	88 (44.9%)	0.772
yes	1407 (57.7%)	299 (57.2%)	108 (55.1%)	
**Monoclonal antibodies**	n=2440	n=523	n=196	
no	2382 (97.6%)	509 (97.3%)	192 (98.0%)	0.865
yes	58 (2.4%)	14 (2.7%)	4 (2.0%)	
**Regimen**	n=2440	n=523	n=196	
PI+dex+/- alkylator	1016 (41.6%)	220 (42.1%)	86 (43.9%)	**0.022**
PI+IMiD+dex	798 (32.7%)	198 (37.9%)	76 (38.8%)	
IMiD+dex	568 (23.3%)	91 (17.4%)	30 (15.3%)	
PI+IMiD+MA+dex	41 (1.7%)	10 (1.9%)	2 (1.0%)	
PI+MA+dex	17 (0.7%)	4 (0.8%)	2 (1.0%)	
**Radiotherapy**	n=2440	n=523	n=196	
no	2288 (93.8%)	357 (68.3%)	127 (64.8%)	**<0.001**
yes	152 (6.2%)	166 (31.7%)	69 (35.2%)	
**ASCT**	n=2440	n=523	n=196	
no	1561 (64.0%)	337 (64.4%)	130 (66.3%)	0.806
yes	879 (36.0%)	186 (35.6%)	66 (33.7%)	
**Best response to treatment**	n=2098	n=424	n=156	
sCR/CR	380 (18.1%)	90 (21.2%)	24 (15.4%)	0.059
VGPR	786 (37.5%)	178 (42.0%)	67 (42.9%)	
PR	663 (31.6%)	110 (25.9%)	46 (29.5%)	
MR	130 (6.2%)	26 (6.1%)	8 (5.1%)	
SD	88 (4.2%)	8 (1.9%)	4 (2.6%)	
PD	51 (2.4%)	12 (2.8%)	7 (4.5%)	

[1] described by absolute and relative frequencies for categorical variables

[2] p-value of Fisher's exact test for categorical variables or Kruskal-Wallis test for continuous variables
Abbreviations: PI (Proteasome inhibitors), IMiD (Immunomodulatory Drugs), MA (Monoclonal Antibodies), dex (dexamethasone or equivalent corticosteroids), ASCT (Autologous Stem Cell Transplant), sCR/CR (stringent Complete Response/Complete Response), VGPR (Very Good Partial Response), PR (Partial Response), MR (Minor Response), SD (Stable Disease), PD (Progressive Disease)
